# A longitudinal study on anaemia in children with *Plasmodium falciparum* infection in the Mount Cameroon region: prevalence, risk factors and perceptions by caregivers

**DOI:** 10.1186/1471-2334-13-123

**Published:** 2013-03-05

**Authors:** Irene Ule Ngole Sumbele, Moses Samje, Theresa Nkuo-Akenji

**Affiliations:** 1 Department of Zoology and Animal Physiology, Faculty of Science, University of Buea, P. O. Box 63, Buea, Cameroon; 2 Department of Medicine, Faculty of Health Sciences, University of Bamenda, P. O. Box 39, Bambili, Cameroon; 3 Department of Microbiology and Parasitology, Faculty of Science, University of Buea, P. O. Box 63, Buea, Cameroon

**Keywords:** Falciparum malaria, Anaemia, Risk factors, Haemoglobin, Prevalence, Children, Caregivers

## Abstract

**Background:**

In heavily endemic malaria areas, it is almost inevitable that malarial infection will be associated with anaemia, although malaria may not be the prime cause of it. Anaemia is a major public health problem in Cameroon. We hypothesized that, factors other than falciparum malaria account for anaemia in the study area.

**Methods:**

A longitudinal study was conducted among 351 *Plasmodium falciparum* positive children to determine the prevalence, risk factors and the perception of anaemia by the caregivers in a semi-rural community. The investigative methods included the use of a structured questionnaire, clinical evaluation and laboratory investigations.

**Results:**

At enrolment the overall prevalence of anaemia as assessed by Hb concentration (Hb < 11 g/dl) was 80.3% (282). Following treatment the prevalence of persistent anaemia was 6% and 46.2% of the children achieved haematological recovery by day 42. Exploratory multiple linear regression analysis showed the following; parasitaemia density (P < 0.01), enlarged spleen (P < 0.05), duration of fever > 2 days (P < 0.01), high white blood cell count (P < 0.001), sex (P < 0.05), iron status indicators (ferritin and transferrin) (P < 0.001), level of education of the caregiver (P < 0.05), management of onset of malaria by caregiver (P < 0.005) and wasting (P < 0.05) to be risk factors for anaemia in children with falciparum infection. Approximately 75.5% (265) of the caregivers had some knowledge about anaemia.

**Conclusion:**

The identified risk factors revealed the important contributors to the pathogenesis of anaemia in the Mount Cameroon region. Control efforts should therefore be directed towards proper health education emphasizing on proper health seeking behaviour and attitudes of the population.

## Background

With about 216 million cases of malaria and an estimated 3.3 billion people reported at risk, malaria is still a major public health challenge [1]. Greater than 80% of malaria-related morbidity and mortality occurs in sub- Saharan Africa due to infections with *Plasmodium falciparum*[2]. In heavily endemic malaria areas, it is almost inevitable that malarial infection will be associated with anaemia, although malaria may not be the prime cause of it [3,4].

Anaemia an indicator of both poor nutrition and poor health is a common and sometimes serious complication of *P. falciparum* infection [5,6]. Anaemia impairs normal development in children and it constitutes a major public health problem in young children in the developing world with wide social and economic implication [7].The highest prevalence of anaemia exists in the developing world where its causes are multi-factorial [8]. The complex aetiology of anaemia involves the interaction between multiple factors including nutritional deficiencies, genetic red blood cell disorders, infectious diseases particularly malaria, hookworm and human immunodeficiency virus infections [9].

Data on the prevalence of anaemia are limited in this area. Cornet *et al*. [7] reported that 42% of the children less than three years of age in Southern Cameroon were anaemic. Furthermore, a 2004 national survey on anaemia in preschool children (< 5 years) revealed anaemia to be a severe public health problem in Cameroon with a prevalence of 68.3% [6]. Information on anaemia prevalence must be collected in order to assess the impact of interventions, the adequacy of strategies implemented, and the progress made in the fight against anaemia. However, estimates of anaemia prevalence by themselves are only useful if they are associated with a picture of the various causal factors that contribute to the development of anaemia in a specific setting [6].

One strategy for reducing the morbidity and mortality that may be associated with anaemia is to identify the predictors that can be readily recognized to facilitate prompt therapeutic interventions especially in children residing in resource-poor settings. Family size, residence, younger age, duration of illness, a palpable spleen or liver, gender, history of fever, pale body, general body weakness, diarrhoea, soil-eating, stunting, malaria parasitaemia and recrudescent infections have been associated with mean haemoglobin levels [10-15]. The factors which identify patients at risk of developing anaemia during malaria have been infrequently studied in this region. It is unclear whether these factors alone or in addition to others, are associated with the anaemia of uncomplicated malaria infection in Cameroonian children. We hypothesized that, factors other than falciparum malaria account for anaemia in the study area.

## Methods

### Study area and subjects

The study was carried out in Muea village a semi rural setting in the Mount Cameroon Region. The study area has been described in detail by Sumbele *et al*. [16]. The children who participated in the study weighed > 5 kg, were ≤ 14 years old, *P. falciparum* parasitaemia positive and sickle test negative. Children who presented with fever, joint pains, headache, malaise, abdominal pain, nausea and vomiting were considered to be symptomatic (clinical malaria). Following government guidelines, children with clinical malaria and malaria parasite positive slides were treated with a standard dose of 4 mg/kg body weight (bw) of Artesunate and 10 mg/kg bw of amodiaquine given once a day for 3 days.

### Study design

This longitudinal study was carried out from January to November 2006, to include the rainy season (April-September) which has been reported as the peak malaria transmission period in the locality [17]. The investigation methods included the use of a structured questionnaire, clinical evaluation and laboratory investigations. Clinical and parasitological examinations were done at enrolment followed by treatment on day zero (D0). Follow up investigations were conducted on D 7, 14, 21, 28 and 42 for children with clinical malaria. At each follow-up visit the children were assessed clinically and fresh samples of capillary blood obtained from them by finger-prick for the determination of haemoglobin (Hb), haematocrit and parasitological counts. Persistent anaemia was defined as Hb concentration that remained below 11 g/dL for the duration of the follow up while haematological recovery was defined as an Hb concentration of at least 11.0 g/dL on D42 in a patient found anaemic on D0 [12,18].

### Questionnaire

A structured questionnaire on the risk factors was administered to caregivers (parents or guardian) in their home settings to obtain information on child and household demographics; treatment seeking behaviour; knowledge, attitude and practices on anaemia; mosquito avoidance practices; anti-malarial drug use and information on type of food consumed in the previous seven days.

### Clinical evaluation

Clinical evaluation was carried out by trained medical personnel from the Muea health centre in their homes and this consisted of general examination. Axillary body temperature was measured using a digital thermometer. A child was considered febrile when he/she had an axillary body temperature ≥ 37.5°C. Weight and height were measured using a Terraillon weighing scale (Terraillon, Paris) and a measuring tape, respectively. Ages of the children were obtained from their mothers and verified from their birth certificates. Height-for-age (HAZ), weight-for-age (WAZ) and weight-for-height (WHZ) standard deviation (SD) scores (z scores) were computed based on the National Center for Health Statistics (NCHS)-WHO growth reference curves using the nutrition module of the Epi Info 2000 programme [2]. Children with <−2 and <−3 SD were classified as malnourished and severely malnourished respectively. The tip of the spleen was felt by pressing the abdomen under the left coastal border and splenomegaly was graded according to the classification of Hackett [19].

### Sample collection

On D0 and D42 approximately 4–5 ml of blood sample was collected from the children by venipuncture into 5 ml sterile disposable syringes (Cathy Yougo) and dispensed into micro-containers or vacutainers containing ethylenediaminetetraacetate (EDTA) solution. Drops of whole blood were dispensed immediately on slides to prepare blood films. The caregivers and their children were instructed clearly on how to collect stool samples. Each child was then given a labelled, clean, wide-mouthed and screw-capped container to bring the stool sample. Labelled blood and stool samples were then transported on ice in a cool box to the University of Buea malaria research laboratory for further analyses.

### Laboratory methods

The thick and thin blood smears prepared on glass slides at the time of blood sampling were stained with Giemsa stain and examined following standard protocols [20]. Parasite density was determined on the bases of number of parasites per 200 leukocytes on thick blood film with reference to subjects white blood cell counts (WBC). If gametocytes were seen, the count was extended to 500 leukocytes [21]. Haemoglobin (Hb) concentration was measured in the field using a Stanbio STAT-Site^R^ Test Kit (STAT-site M^Hgb^ Meter, stanbio Laboratory, Texas, USA) following the manufacturer’s instructions. Anaemia was defined as Hb concentration < 11 g/dL and further categorized as mild (Hb between 10.1–10.9 g/dl), moderate (Hb between 7.0 -10.0 g/dl) and severe (Hb < 7 g/dl) anaemia [20,22]. White blood cell (WBC) counts were determined using the improved Naubauer haemacytometer as described by Cheesbrough [20] and Dacie and Lewis [23]. Determination of platelet count was done using the improved Naubauer ruled counting chamber as described by Cheesebrough [20].

Stool samples were examined for helminths by the Kato–Katz thick smear technique [20]. Plasma iron concentration was assayed using the iron ferrozine reagent (Ferrozine®, Elitech, France) following manufacturer’s instruction. Plasma transferrin was assayed by radial immunodiffussion using the transferrin id reagent kit (Cromatest, Linear chemicals S.L) following the manufacturer’s instruction. Plasma ferritin was assayed using the LISA 300 plus system (Labo Tech Medicale) and ferritin calibrator set (Human Gesellschaft fur Biochemica und Diagnostica mbH, Germany) prepared from human sera. Iron deficiency was defined as plasma ferritin concentration < 30 ng/mL.

### Statistical analysis

Data was doubly entered and validated using SPSS version 11.5 and Epi-info soft ware. Analysis was done with SPSS version 17 (SPSS, Inc., Chicago, IL, USA). Data was summarized into means and standard deviations, and percentages were used in the evaluation of the descriptive statistics. Chi square (χ2) test was used to compare the prevalence of anaemia in the various groups of subject. The Epi Info package (Epi Nut module) was used to analyze WAZ, WHZ, and HAZ z-scores. Four exploratory multiple linear regression (MLR) models namely; biological (host and parasite factors ), socio economic, management and prevention and integrated models were created containing all the possible associations with haemoglobin as the dependent variable to assess the factors associated with the risk of developing anaemia in children with falciparum malaria. Exploratory analyses were used to reduce the number of potential confounders. The exploratory MLR model was run using the z scores of the variables. All results were considered to be statistically significant at 95% probability level (P < 0.05).

### Ethical consideration

The study received administrative approval by the Ministry of Public Health Cameroon. The Ethical Committee of the University of Buea issued the ethical clearance document. Additional authorisation was obtained from the local health committee and the village chief. Children participated in the study if a parent or guardian signed the informed consent form. The parents or guardian and their children were informed that their participation in the study was voluntary and could withdraw at any time without any explanation.

## Results

### Baseline characteristics of the subjects

A total of 351 *P. falciparum* infected children (46.2% females and 53.8% males) with a mean age of 6.45 ± 7.9 years (6 months to 14 years) residing in the Mount Cameroon region were evaluated for the prevalence and risk factors of anaemia. More than half of the children were infected with soil-transmitted helminths (STH) and the prevalence of fever and malnutrition was low (Table 1). The most prevalent STH was *Ascaris lumbricoides* (49.0%) with a mean egg per gram of faeces of 549.5 ± 2008.8, followed by *Trichuris trichiura* (43.4%, 119.6 ± 167.6) and hookworms (19.4%, 40.3 ± 63).

**Table 1:** **Baseline characteristics of the 351 children with *P. falciparum *infection**

**Parameter**		**All**
Age groups	≤ 5 years % (n)	51.3% (180)
	> 5 years % (n)	48.7% (171)
Sex	Female % (n)	46.2% (162)
	Male % (n)	53.8% (189)
Mean age in years (n)		6.45 ± 7.9 (351)
Mean height in cm (Range)		107.40 ± 23.18 (59 – 170)
Mean weight in kg (Range)		21.09 ± 9.99 (7–55)
“Prevalence (%) of fever (Temp. ≥ 37.5°C)” (n)		29.3 (103)
Mean temp in °C. (Range)		37.16 ± 0.64 (35.5 - 40.4)
Mean Hb in g/dl (Range)		9.42 ± 1.75 (5.9 – 15.9)
Mean egg per gram of faeces (Range)		285.67 ± 1310.46 (24–14976)
Mean WBC ×10^9^/L (Range)		11.64 ± 19.39 (2.2 – 33.5)
Geometric mean parasite density (Range)		795.1 ± 18511.9 (40 – 202660)
Prevalence (%) of clinical malaria (n)		53.0 (186)
Prevalence (%) of gametocytaemia (n)		29.9 (105)
Prevalence (%) of splenomegaly (n)		25.1 (88)
Prevalence (%) of soil-transmitted helminth infection(n)		55.8 (196)
Prevalence (%) of malnutrition (n)		23.4 (82)

Stunting, wasting and underweight were prevalent in 20.5%, 2.6% and 8.1% of the children while 6.9%, 0.3%, and 1.4% were severely stunted, wasted and underweight respectively. Children who were ≤ 5 years old had a significantly (χ^2^ = 4.9, P = 0.02) higher prevalence of underweight (11.2%) when compared with those > 5 years (4.7%). Equally, malnutrition was significantly (χ^2^ = 5.01 P = 0.03) higher in males (28.0%) than in females (17.9%).

The prevalence of iron deficiency was comparable (P > 0.05) in males (27.5%) and females (27.8%). Although not statistically significant (P > 0.05), the prevalence of abnormal transferrin, ferritin and iron values was higher in females (60.5%, 64.5%, 57.9%) than males (51.1%, 57.6% and 55.9% respectively).

### Prevalence of anaemia at enrolment

Anaemia as assessed by Hb concentration (Hb < 11 g/dl) was prevalent in 80.3% (282) of the children. The prevalence of mild, moderate and severe anaemia in the study population was, 22.7% (64), 65.2% (184) and 12.1% (34) respectively. Children ≤ 5 years had a significantly (P < 0.01) higher prevalence of anaemia when compared with those greater > 5 years. Similarly the prevalence of anaemia in children with enlarged spleens was significantly (P < 0.01) higher than those with normal spleen (Table 2). The prevalence of anaemia in children with fever increased with the category of pyrexia with all children having hyper pyrexia (Temp ≥ 39°C) being anaemic (Table 2). Although not significant (P > 0.05) children who were co-infected with helminths, had clinical malaria and those malnourished had a higher prevalence of anaemia than their counterparts (Table 2).

**Table 2:** **Anaemia prevalence in the various categories of children with *P. falciparum *infection**

**Description**		**N**	**Anaemia prevalence in % (n)**	**Level of significance**
Sex	Female	162	79.0 (128)	χ^2^ = 0.34
	Male	189	81.5 (154)	P = 0.56
Age group	≤ 5 years	180	87.2 (157)	χ^2^ = 11.07
	> 5 years	171	73.1 (125)	P = 0.001^b^
Malaria status	Clinical malaria	186	83.3 (155)	χ^2^ = 2.24
	Asymptomatic	165	77.0 (127)	P = 0.09
Pyrexia category	Normal (Temp. ≤ 37.4°C)	248	76.6 (190)	χ^2^ = 7.98
	Mild pyrexia (Temp. 37.5 - 38.9°C)	96	88.5 (85)	P = 0.02^a^
	Hyper pyrexia (Temp ≥ 39°C)	7	100 (7)	
Parasite density category	Parasite /μl of blood ≤ 5000	309	79.6 (246)	χ^2^ = 0.87
	Parasite /μl of blood > 5000	42	85.6 (36)	P = 0.35
Gametocytaemia	Positive	105	86.7 (91)	χ^2^ = 3.8
	Negative	246	77.6 (191)	P = 0.05
Spleen size	Enlarged	88	92.0 (81)	χ^2^ = 10.19
	Normal	263	76.4 (201)	P = 0.001^b^
History of fever	≥ 2 days	66	90.9 (60)	χ^2^ = 5.75
	< 2 days	76	77.6 (59)	P = 0.06
	No fever	209	78.0 (163)	
Helminth status	Positive	196	83.7 (164)	χ^2^ = 0.26
	Negative	155	80.6 (125)	P = 0.61
Nutritional status	Malnourished	82	82.9 (68)	χ^2^ = 0.45
	Normal	269	79.6 (214)	P = 0.50

^a^ significant at P < 0.05 level, ^b^ significant at P < 0.01 level.

### Prevalence of anaemia and clinical malaria posts treatment

**Figure 1:**
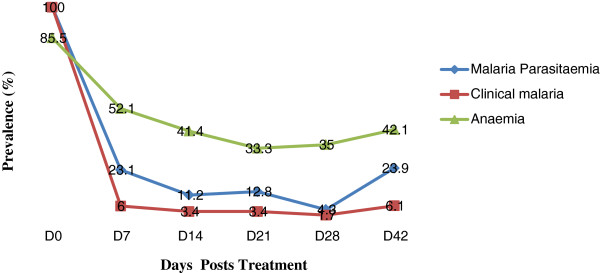
Prevalence of malaria parasitaemia, clinical malaria and anaemia during follow up.

**Figure 2:**
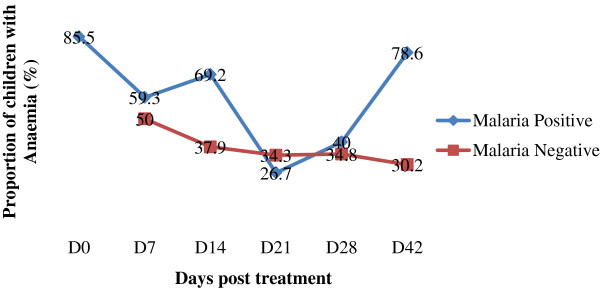
Proportion of children with anaemia during follow up.

Out of the 186 children with clinical malaria who were treated with Artesunate –Amodiaquine, 138 children gave their assent/consent to the follow up investigation regime and 117 were successfully followed for 42 days. The prevalence of malaria parasitaemia, clinical malaria and anaemia showed a decline following treatment and the highest decline was observed on D28 (4.3%), D28 (1.7%) and D21 (33.3%) respectively (Figure 1). Children who were malaria parasite positive showed a higher prevalence of anaemia on D14 (69.2%) and D42 (78.6%) than those negative (D14 = 37.9%, D42 = 30.2%) post treatment (Figure 2). This difference in prevalence was statistically significant (D14; χ^2^ = 4.7, P = 0.03 and D42; χ^2^ = 20.2, P < 0.001). Although not significant the prevalence of anaemia was higher in malaria parasite negative children (34.3%) than their positive counterparts (26.7%) on D21 (Figure 2).

### Persistent anaemia and haematological recovery

Persistent anaemia (Hb concentration that remained below 11 g/dl for the duration of the follow-up) was prevalent in 6% (7/117) of the children. The prevalence of persistent anaemia in the different sexes were comparable (P > 0.05) even though male children were 0.9times (95% CI: 0.19 – 4.12) more likely to have persistent anaemia than females. Although not significant (P > 0.05), the prevalence of persistent anaemia was higher in children ≤ 5 years (7.0%) than those > 5 years (1.9%) old.

On D42 post treatment, 46.2% (54/117) of the children (40.7% females and 59.3% males) achieved haematological recovery. Male children were 0.7times (95% CI: 0.4 – 1.4) likely to achieve haematological recovery than their female counterpart. Haematological recovery was comparable (P > 0.05) in children ≤ 5 (39.5%) and those above 5 years (38.5%) of age.

### Identification of risk factors of anaemia

The exploratory MLR was run using the z scores of the variables and the tolerance statistics were all below 1. All the Variance Inflation Factors (VIF) were < 2. The lower values of the condition index (1.084 - 4.478) confirm that the collinearity diagnostic was successful.

### Host and parasite factors associated with haemoglobin concentration

Two biological sub exploratory MLR models were run to examine host and parasite factors associated with Hb concentration. The first sub model examined host and parasite factors associated with haemoglobin concentration while the other examined in addition iron status indicators as risk factors for anaemia. In the first sub model, WBC count (P = 0.001), parasitaemia density (P = 0.002) and length of fever in days (P = 0.001) were significantly (P < 0.01) associated with Hb concentration. Furthermore a significant negative correlation of Hb concentration with parasitaemia density, length of fever in days and WBC count was observed (Table 3). In the second sub exploratory MLR model (R^2^ = 0.43, adjusted R^2^ = 0.37) which included iron indices, the variables with significant influence on the Hb concentration were spleen size (β = 0.17, P = 0.02), sex of the child (β = 0.13, P = 0.03), transferrin (β = 0.0.18, P = 0.01) and ferritin (β = 0.38, P = 0.0001) concentrations.

**Table 3:** Multilinear regression model showing some host and parasite factors associated with anaemia

**Variable**	**β value**	**P value**	**Correlation**
			**Partial**
Age	−0.037	0.430	−0.039
Sex	0.037	0.434	0.039
Temperature	−0.078	0.118	−0.078
WBC	−0.186	0.0001^c^	−0.192^c^
Parasitaemia density	−0.153	0.002^b^	−0.155^b^
Gametocytaemia	−0.082	0.083	−0.086
Length of fever (days)	−0.162	0.001^c^	−0.164^c^
R square	0.128		
Adjusted R square	0.113		

^b^ Significant at P < 0.01 level, ^c^ significant at P < 0.001 level.

### Socio-economic factors associated with haemoglobin concentration

In the socio economic exploratory MLR model (R^2^ = 0.06 and adjusted R^2^ = 0.01) only the level of education of the guardian/caregiver was identified as a risk factor (P = 0. 04) for anaemia. Children whose caregivers were illiterate had a higher prevalence of anaemia (90%) when compared with those whose caregivers had basic education (77. 8%).

### Factors associated with management and prevention of malaria

The caretaker management ability of malaria (when a caretaker decides to take the child to a health facility) was the only factor significantly (P = 0.005) associated with the Hb concentration in this model (R^2^ = 0.05, adjusted R^2^ = 0.03). Anaemia was more prevalent in those who used other traditional methods of management of malaria (90.2%) than in those who were taken to the hospital as soon as they took ill (73.0%) or those who went to a health facility only after other treatment(s) failed (81.1%).

### Integrated values mapping (IVM) to identify predictors of haemoglobin concentration

Given the poor predictive power and sensitivity of the socio-economic, management and prevention of malaria model, the IVM model incorporated only the biological and some aspects of the malaria prevention model. Based on the IVM model splenomegaly (P < 0.05), wasting (P < 0.05), sex (P < 0.05), transferrin (P < 0.01) and ferritin (P < 0.001) concentrations, were significantly associated with haemoglobin concentration in children with falciparum infection (Table 4).

**Table 4:** An integrated model identifying factors associated with anaemia

**Variables**	**β value**	**P value**	**Correlations**
			**Partial**
Use of bednets	−0.093	0.298	−0.097
Caretaker management of malaria	−0.093	0.228	−0.112
Egg per gram of feaces	−0.019	0.789	−0.025
Temperature	−0.076	0.349	−0.087
Platelet/L	−0.025	0.744	−0.031
WBC × 10 ^9^/L	−0.054	0.485	−0.065
Length of fever (days)	0.037	0.651	0.042
Parasitaemia density	0.047	0.549	0.056
Transferrin /mg/dl)	0.231	0.002^b^	0.283^b^
Ferritin /ng/ml)	0.385	0.0001^c^	0.432^c^
Iron (ug/dl)	0.089	0.238	0.110
Gametocytaemia	−0.056	0.437	−0.073
Splenomegaly	0.203	0.018^a^	0.218^a^
WAZ (under weight)	−0.004	0.967	−0.004
HAZ (stunting)	−0.052	0.541	−0.057
WHZ (wasting)	0.189	0.042^a^	0.189^a^
Age	−0.020	0.823	−0.021
Sex	0.168	0.025^a^	0.207^a^
R square	0.497		
Adjusted R square	0.388		

^a^ significant at P< 0.05 level, ^b^ Significant at P < 0.01 level, ^c^ significant atP < 0.001 level.

### Perception of anaemia by caregivers of the children

Approximately 75.5% (265) of the caregivers had some knowledge about anaemia and the majority (91.3%) of them were cognisant of pallor as the only symptom of anaemia. If and when a respondent discovered the child was anaemic, only 36.1% of them were likely to consult a physician while the rest did self medication. While 16.5% of the caregivers will self medicate with any available iron tablet, 83.5% of them used herbal remedies. About 63.9% of the respondents were aware of iron containing foods of which vegetables (57.3%) and plantains (25.9%) were the foods of choice. A diet recall in the previous seven days showed fish, plantain, huckleberry and red pepper to be frequently consumed foods.

## Discussion

Anaemia is a major public health problem in Cameroon. In addition to measuring the haemoglobin concentration which is the most reliable indicator of anaemia at the population level [6], the causes of anaemia need to be identified as they may vary according to the population. The high prevalence of anaemia (80.3%) observed in the study population is comparable to the 82% obtained by Jourdan *et al*. [24] in children attending a clinic in Northern Cameroon and the > 70% obtained by Desai *et al*. [13] in pre -school children in Kenya. This is not unexpected in a semi rural community where malaria is hyperendemic [17] and majority of the household heads are farmers with no steady source of income [16]. Furthermore Mogensen *et al*. [22] reported 30%–90% of children to be anaemic at any time in malaria endemic areas of sub-Saharan Africa.

Following treatment more than half of the children (63/117) never achieved haematological recovery. In line with Obonyo *et al*. [18] haematological recovery in the children was associated with the clearance of parasitaemia. While the occurrence of clinical malaria after day 28 prevented haematological recovery in some of the children, the presence of persistent anaemia in children who were clinically and parasitologically cured suggests factors other than malaria may be involved in the pathogenesis of anaemia in the population.

While our findings showed a significant negative correlation (P < 0.01) between haemoglobin level and parasite density, others [7,25] failed in finding such a correlation, even though they identified malaria as a risk factor for anaemia. Kahigwa *et al*. [26] reported *P. falciparum* parasitaemia to be the single most important factor associated with anaemia. Hyperparasitaemia has been considered as one of the possible manifestations of severe and complicated malaria, depending on the endemic area [27]. Anaemia in acute falciparum malaria is caused by increased destruction of both infected and non-infected erythrocytes and decreased erythropoiesis. However, we observed that even the very low density infections were associated with moderate anaemia in the children.

In addition to the density of parasitaemia, hypersplenism is also thought to contribute to the early anaemia of acute malaria through sequestering red blood cells in the spleen [28]. Our studies confirm this finding since a significant difference in the prevalence of anaemia was observed in children with enlarged spleens (92.0%) when compared with those having normal spleen sizes (76.4%). Children with a palpable spleen were 4.04 (95% CI: 1.79 – 9.08) times at risk of presenting with anaemia than those with normal spleens. The spleen is a key site for removal of parasitized red blood cells, generation of immunity and production of new red blood cells during malaria [29].

Protracted infections with malaria parasite are associated with clinically significant RBC destruction [12]. Children with length of fever ≥ 3 days were significantly at risk of developing anaemia. Contrary to Ong’echa *et al*. [30] and Grenfell *et al*. [31] who reported fever not to be associated with haemoglobin concentration, our findings revealed a significant negative correlation (r = − 0.18, P < 0.01) between haemoglobin concentration and temperature and a direct relationship between temperature and parasitaemia with the fever rate decreasing with increased age. The observed negative association between temperature and haemoglobin may be due to certain immunologic responses such as the secretion of high levels of tumour necrosis factor-α (TNF-α), a potent pyrogen. Chronic low grade production of TNF- α, in response to *P*. *falciparum* parasitaemia may induce dyserythropoiesis thus contributing to the pathogenesis of malarial anaemia [32]. However, a timely evaluation of all febrile illness, case-recognition and use of appropriate antimalarial therapy are indispensable to malaria and anaemia control in order to optimize clinical outcomes [17].

We found a significant inverse association between WBC count and haemoglobin concentration. Studies by Ladhani *et al*. [33] associated a high WBC count with severe anaemia. While normal total WBC count has been associated with malaria infection [34], leucopoenia appears to be a common finding in both non-immune patients with falciparum malaria and semi-immune children living in malaria-endemic regions, where WBC may be as low as 1-2×10^9^/L [3]. In view of the fact that our study included children with different degrees of malaria with a WBC count ranging from 2.2 – 33.5 ×10^9^/L, it may be possible that some of these children had concurrent bacterial infections which were not clinically apparent and were beyond the scope of this study. Nonetheless bacteraemia most commonly due to nontyphoid salmonella, has been strongly associated with severe anaemia [35,36].

Although no significant difference was observed in the prevalence of anaemia between the different sexes, anaemia was higher in males than in females. Males were 0.84 (95% CI: 0.59 – 1.20) times more likely to be anaemic than females. Similar observations were made in Kenya [30,37], Tanzania [38] and Ghana [39]. This may be attributed to the fact that males were significantly more malnourished than females. Also male children were 1.4 times (95% CI: 0.85 -2.1) at risk of carrying gametocytes than their female counterpart. The higher gametocyte carriage observed in males (32.8%) when compared with females (26.5%) may have exacerbated a decrease in the haemoglobin concentration. Even though the MLR model did not significantly identify gametocyte carriage as a risk factor of anaemia, the prevalence of anaemia was higher in children who were gametocyte positive than those negative.

Ferritin and transferrin were significantly associated with haemoglobin concentration in the IVM model. Further observations highlighted a significant difference in the prevalence of anaemia between children with abnormal (96.6%; 92.5%) and normal (59.8%; 70.3%) ferritin and transferrin values respectively. Stoltzfus *et al*. [38] reported the strongest relationship of serum ferritin and haemoglobin to occur in children who were malaria free and < 30 months of age. The usefulness of transferrin in monitoring infections has been controversial and the behaviour of ferritin as an acute phase reactant may obscure the relationship of haemoglobin to iron stores. However, a large proportion of anaemia in this population could not be explained by iron deficiency.

Our study revealed the level of education of the caregiver to be a significant determinant of haemoglobin concentration in children with falciparum malaria in this semi rural setting of the mount Cameroon region. Furthermore a negative trend (r = − 0.16) in the relationship between haemoglobin concentration and education level was observed. Children whose caregivers were illiterate showed a high prevalence of anaemia (90%), when compared with those whose caregivers had basic education (77.8%). Correlation between education level and anaemia has also been reported by Kahigwa *et al*. [26] in Tanzania, Ong’echa *et al*. [30] in Western Kenya and Al-Mekhlafi *et al*. [40] in Malaysia. This may be linked to their having knowledge about anaemia and iron containing foods as our findings showed a significant difference in the knowledge of anaemia between those with basic education (> 76.5%) and those illiterate (20.0%).

The significant association of the caretaker management ability of malaria and haemoglobin concentration may be linked to the effect of protracted febrile infection with the malaria parasite on the red blood cells. Pre-hospital antimalarial treatment of febrile children by caregivers/parents with mostly traditional herbs or drugs of questionable quality remains a significant common practice among individuals in the population as revealed by the questionnaire survey. Lack of proper education and poverty may be contributing factors to these attitudes and practices. In a previous related study [17], early treatment with effective antimalarial was demonstrated to decrease the morbidity and mortality due to malaria.

Wasting a manifestation of acute malnutrition [41] was significantly associated with the haemoglobin concentration as indicated by the IVM model. Although the prevalence of wasting (2.6%) in the children with falciparum malaria was low, 8 out of the 9 children (88.9%) were anaemic. Correlation between haemoglobin and nutritional status has also been reported by Nabakwe & Ngare [42]. Ehrhardt *et al*. [43] reported malnutrition to be a fundamental factor contributing to malaria-associated morbidity and anaemia, even if the latter exhibits multifactorial patterns. The high prevalence of anaemia coupled with the presence of malnutrition may have contributed to impaired growth in the children as weight significantly positively correlated with haemoglobin levels (r = 0.11, P = 0.03). Nutritional inadequacies causing stunting and underweight may also impair host immunity, further exacerbating the effects of malaria [41,44]. However, improving the nutritional status of the children may lessen the morbidity due to falciparum malaria.

Even though the majority of caregivers were aware of pallor as a symptom of anaemia, none could detect whether the child was anaemic before assessment of haemoglobin concentration. The efficiency and applicability of pallor examination in the detection of anaemia is important in clinical circumstances [22]. Signs of pallor may be used as a tool to detect moderate (or severe) anaemia with sensitivities and specificities around 60%–86% [45]. The inability of caregivers to diagnose the paleness of the conjunctiva and palms which were apparent in those with severe anaemia (12.1%), confirms earlier findings [22] that assessment of pallor depends on training.

## Conclusions

In addition to malaria, enlarged spleen, duration of fever > 2 days, high white blood cell count, sex, iron status indicators (ferritin and transferrin), level of education of the caregiver, management of onset of malaria by caregiver and wasting are important contributors to the pathogenesis of anaemia in the Mount Cameroon region. Improving the case management of malaria is likely to reduce the burden of anaemia hence useful health benefits. Control efforts should therefore be directed towards proper health education emphasizing on proper health seeking behaviour and attitudes of the population. In addition to malaria which is commonly considered to be a principal cause of anaemia additional diagnoses including malnutrition and helminths should be considered.

## Endnotes

^a^Significant at P < 0.05 level, ^b^Significant at P < 0.01 level, ^c^significant at P < 0.001 level.

## Abbreviations

D: Day; HAZ: Height-for-age; Hb: Haemoglobin; IVM: Integrated values mapping; MLR: Multiple linear regression model; STH: Soil-transmitted helminths; WBC: White blood cell; WAZ: Weight-for-age; WHZ: Weight-for-height

## Competing interest

We the authors declare that we have no competing interests.

## Authors’ information

IUNS: PhD and Lecturer of Parasitology, Department of Zoology and Animal Physiology.

MS: MSc and Assistant lecturer of Biochemistry, Department of Medicine.

TNA: PhD and Professor of Medical Parasitology, Department of Microbiology and Parasitology, Dean Faculty of Science.

## Authors’ contribution

IUNS was involved in all phases of the study, including study design, data collection, data analysis, interpretation, and write-up of the manuscript; TNA. designed and supervised the study and also revised the manuscript. MS was involved in the collection and laboratory examination of samples. All authors read and approved the final manuscript.

## Pre-publication history

The pre-publication history for this paper can be accessed here:

http://www.biomedcentral.com/1471-2334/13/123/prepub

## References

[B1] WHO World Malaria report. Global malaria programme 2011 Geneva: World Health Organization publication

[B2] WHO World malaria report 2005 Geneva: World Health Organization/United Nations Children’s Fund http://rbm.who.int/wmr2005/

[B3] Facer CA Hematological aspects of malaria Infection and Hematology 1994 Oxford: Butterworth Heinmann Ltd 259 294

[B4] Murphy GS Oldfield EC Falciparum malaria Infect Dis Clin North Am 1996 10 747 770 10.1016/S0891-5520(05)70325-1 8958167

[B5] Nussenblatt V Semba RD Micronutrient malnutrition and the pathogenesis of malarial anaemia Acta Trop 2002 82 321 337 10.1016/S0001-706X(02)00049-9 12039672

[B6] Benoist B, McLean E, Egli I, Cogswell M Worldwide prevalence of anaemia 1993–2005: WHO global database on anaemia 2008 Geneva: World Health Organization publication

[B7] Le Cornet M Hesran JUL Fievet N Personne P Gounoue R Beyeme M Deloron P Prevalence of and risk factors for anaemia in young children in southern Cameroon Am J Trop Med Hyg 1998 58 606 611 9598449 10.4269/ajtmh.1998.58.606

[B8] Tolentino K Friedman FF An update on anaemia in less developed countries Am J Trop Med Hyg 2007 77 44 51 17620629

[B9] van Eijk AM Ayisi JG ter Kuile FO Misore A Otieno JA Kolczak MS Kager PA Steketee RW Nahlen BL Malaria and human immunodeficiency virus infection as risk factors for anemia in infants in Kisumu, western Kenya AmJTrop Med Hyg 2002 67 44 53 10.4269/ajtmh.2002.67.44 12363063

[B10] Salako LA Ajayi FO Sowunmi A Walker O Malaria in Nigeria: a revisit Ann Trop Med Par 1990 84 435 445 10.1080/00034983.1990.11812493 2256767

[B11] Luxemburger C Thwai KL White NJ Webster HK Kyle DE Maelankirri L Chongsuphajaisiddhi T Nosten F The epidemiology of malaria in a Karen population on the western border of Thailand Trans R Soc Trop Med Hyg 1996 90 105 111 10.1016/S0035-9203(96)90102-9 8761562

[B12] Price RN Simpson JA Nosten F Luxemburger C Hkirjaroen L ter Kuile F Chongsuphalaisiddh T White NJ Factors contributing to anaemia after uncomplicated falciparum malaria Am J Trop Med Hyg 2001 65 614 622 11716124 10.4269/ajtmh.2001.65.614 PMC4337986

[B13] Desai MR Terlouw DJ Kwena AM Phillips-Howard PA Kariuki SK Wannemuehler KA Odhacha A Hawley WA Shi YP Nahlen BL Ter Kuile FO Factors associated with hemoglobin concentrations in pre-school children in Western Kenya: cross-sectional studies Am J Trop Med Hyg 2005 72 47 59 15728867

[B14] Ronald LA Kenny SL Klinkenberg E Akoto AO Boakye I Barnish G Donnelly MJ Malaria and anaemia among children in two communities of Kumasi, Ghana: a cross-sectional survey Mal J 2006 5 105 10.1186/1475-2875-5-105 PMC1654171 17094806

[B15] Zhao A Zhang Y Peng Y Li J Yang T Liu Z Yanli L Wang P Prevalence of anemia and its risk factors among children 6–36 months old in Burma Am J Trop Med Hyg 2012 87 306 311 10.4269/ajtmh.2012.11-0660 22855763 PMC3414569

[B16] Sumbele IUN Nkuo-Akenji T Samje M Ndzeidze T Ngwa EM Titanji VPK Haematological changes and recovery associated with treated and untreated *Plasmodium falciparum* infection in children in the Mount Cameroon Region J Clin Med Res 2010 2 143 151

[B17] Nkuo-Akenji T Ntonifor NN Ching JK Kimbi HK Ndamukong KN Anong DA Boyo MG Titanji VPK Evaluating a malaria intervention strategy using knowledge, practices and coverage surveys in rural Bolifamba, South West Cameroon Trans R Soc Trop Med Hyg 2005 99 325 332 10.1016/j.trstmh.2003.12.016 15780338

[B18] Obonyo CO Taylor W Ekvall H Kaneko A Ter Kuile F Olliaro P Bjorkman A Oloo AJ Effect of artesunate plus sulfadoxine-pyrimethamine on haematological recovery and anaemia, in Kenyan children with uncomplicated, *Plasmodium falciparum* malaria Ann Trop Med Parasitol 2007 101 281 295 10.1179/136485907X176337 17524243

[B19] Gilles HM Pathology of malaria: Handbook of malaria infection in the tropics 1997 Italy: Italian Association Amicidi Raoul Follerau (AIFO)

[B20] Cheesbrough M District Laboratory Practice in Tropical Countries. Part1& 2 1998 Edinburg Building UK: Cambridge University Press

[B21] Trape JF Rapid evaluation of malaria parasite density and standardization of thick smear examination for epidemiological investigations Trans R Soc Trop Med Hyg 1985 79 181 184 10.1016/0035-9203(85)90329-3 3890280

[B22] Mogensen CB Sørensen JE Bjorkman A Pallor as a sign of anaemia in small Tanzanian children at different health care levels Acta Trop 2006 99 113 118 10.1016/j.actatropica.2005.12.010 17022931

[B23] Dacie JV Lewis SM Red Cell Count in Practical Haematology 1995 Michigan: Churchill Livingstone

[B24] Jourdan PM Laoussou P Lybie A Kjetland EF Indicators of anaemia in under-fives with malaria at a hospital in Northern Cameroon W Afri J Med 2008 27 7 12 18689296

[B25] Premji Z Hamisi Y Shiff C Minjas J Lubega P Makwaya C Anemia and *Plasmodium falciparum* infections among young children in an holoendemic area, Bagamoyo, Tanzania Acta Trop 1995 59 55 64 10.1016/0001-706X(94)00079-G 7785526

[B26] Kahigwa E Schellenberg D Sanz S Aponte JJ Wigayi J Mshinda H Alonso P Menendez C Risk factors for presentation to hospital with severe anaemia in Tanzanian children: a case–control study Trop Med Int Hlth 2002 7 823 830 10.1046/j.1365-3156.2002.00938.x 12358616

[B27] Warrell DA Molyneux ME Beales PF Severe and complicated malaria Trans R Soc Trop Med Hyg 1990 84 1 65 2219249

[B28] Menendez C Fleming AF Alonso PL Malaria - related anaemia Parasitol Today 2000 16 469 476 10.1016/S0169-4758(00)01774-9 11063857

[B29] Engwerda CR Beattie L Amante FH The importance of the spleen in malaria Trends Parasitol 2005 21 75 80 10.1016/j.pt.2004.11.008 15664530

[B30] Ong’echa JM Keller CC Were T Ouma C Otieno RO Landis-Lewis Z Ochiel D Slingluff JL Mogere S Ogonji GA Orago AS Vulule JM Kaplan SS Day RD Perkins DJ Parasitemia, anemia, and malarial anemia in infants and young children in a rural holoendemic *Plasmodium falciparum* transmission area Am J Trop Med Hyg 2006 74 376 385 16525094

[B31] Grenfell P Fanello CI Magris M Goncalves J Metzger WG Vivas-Martınez S Curtis C Vivas L Anaemia and malaria in Yanomami communities with differing access to healthcare Trans R Soc Trop Med Hyg 2008 102 645 652 10.1016/j.trstmh.2008.02.021 18405929

[B32] Tchinda VHM Tadem AD Tako EA Tene G Fogako J Nyonglema P Sama G Zhoue A Leke RGF Severe malaria in Cameroonian children: correlation between plasma levels of three soluble inducible adhesion molecules and TNF-α Acta Trop 2007 102 20 28 10.1016/j.actatropica.2007.02.011 17397790

[B33] Ladhani S Lowe B Cole AO Kowuondo K Newton CRJC Changes in white blood cells and platelets in children with falciparum malaria: relationship to disease outcome Br J Haematol 2002 119 839 847 10.1046/j.1365-2141.2002.03904.x 12437669

[B34] Bashawri LAM Mandil AA Bahnassy AA Ahmed MA Malaria: hematological aspects Ann S Med 2002 22 372 377 10.5144/0256-4947.2002.372 17146269

[B35] Graham SM Walsh AL Molyneux EM Phiri AJ Molyneux ME Clinical presentation of non-typhoidal Salmonella bacteraemia in Malawian children Trans R Soc Trop Med Hyg 2000 94 310 314 10.1016/S0035-9203(00)90337-7 10975008

[B36] Bronzan RN Taylor TE Mwenechanya J Tembo M Kayira K Bwanaisa L Njobvu A Kondowe W Chalira C Walsh AL Phiri A Wilson LK Molyneux ME Stephen M Graham SM Bacteremia in Malawian children with severe malaria: prevalence, etiology, HIV coinfection, and outcome J Infect Dis 2007 195 895 904 10.1086/511437 17299721

[B37] Brooker S Peshu N Warn PA Masobo M Guyatt H Marsh K Snow RW The epidemiology of hookworm infection and its contribution to anaemia among pre-school children on the Kenyan coast Trans R Soc Trop Med Hyg 1999 93 240 246 10.1016/S0035-9203(99)90007-X 10492749

[B38] Stoltzfus RJ Chwaya HM Montresor A Albonico M Savioli L Tielsch JM Malaria, hookworms and recent fever are related to anaemia and iron status indicators in 0-5-y old Zanzibari children and these relationships change with age J Nutri 2000 130 1724 1733 10.1093/jn/130.7.1724 10867043

[B39] Owusu-Agyei S Fryauff DJ Chandramohan D Koram KA Binka FN Nkrumah FK Utz GC Hoffman SL Characteristics of severe anemia and its association with malaria in young children of the Kassena-Nankana District of Northern Ghana Am J Trop Med Hyg 2002 67 371 377 12452491 10.4269/ajtmh.2002.67.371

[B40] Al-Mekhlafia MH Surina J Atiyab AS Ariffinc WA Mahdya AKM Abdullah HC Anaemia and iron deficiency anaemia among aboriginal schoolchildren in rural peninsular Malaysia: an update on a continuing problem Trans R Soc Trop Med Hyg 2008 102 1046 1052 10.1016/j.trstmh.2008.05.012 18617209

[B41] Caulfield LE Richard SA Black RE Under nutrition as an underlying cause of malaria morbidity and mortality in children less than five years old Am J Trop Med Hyg 2004 71 Suppl 2 55 63 15331819

[B42] Nabakwe EC Ngare DK Health and nutritional status of children in Western Kenya in relation to vitamin a deficiency E Afri J Pub Hlth 2004 1 1 5

[B43] Ehrhardt S Burchard GD Mantel C Cramer JP Kaiser S Kubo M Otchwemah RN Bienzle U Mockenhaupt FP Malaria, anemia, and malnutrition in african children—defining intervention priorities J Infect Dis 2006 194 108 114 10.1086/504688 16741889

[B44] Verhoef H West CE Veenemans J Beguin Y Kok FJ Stunting may determine the severity of malaria-associated anaemia in African children Pediatrics 2002 110 1 5 10.1542/peds.110.1.1 12359821

[B45] Luby SP Kazembe PN Redd SC Ziba C Nwanyanwu OC Hightower AW Franco C Chitsulo L Wirima JJ Olivar MA Using clinical signs to diagnose anaemia in African children Bull World Hlth Organ 1995 73 477 482 PMC2486784 7554019

